# *Proceedings B* 2022: the year in review

**DOI:** 10.1098/rspb.2022.2479

**Published:** 2023-01-11

**Authors:** Spencer C. H. Barrett

**Affiliations:** *Editor-in-Chief*

The past 3 years of ‘living with Covid’ have been challenging for scientific publishing, but as I write my year-end review for 2022, there are signs for optimism. *Proceedings B* has recovered well from this difficult period, with monthly submissions creeping upwards in the second half of 2022, and the journal is attracting high-quality submissions and continuing to diversify its content. Staff are now working regularly at 6–9 Carlton House Terrace, and last May we had the first ‘in-person’ editorial board meeting in London for 2 years. The impression I got at the meeting was that both editors and staff were relieved and enthusiastic to be interacting face-to-face without the constraints imposed by the more structured videoconference format we used in 2020 and 2021. A convivial night at a London pub and our traditional meal for staff and editors at a London restaurant provided ample opportunities for ‘chin-wagging’ and reinforced to us all that the social dimension of our work is critical for building relationships, brainstorming and developing new initiatives, several of which have taken shape during the year.

From 1 January to 31 October 2022, we received 2092 submissions, a decrease of 184 in comparison with the same period in 2021. Because 2021 saw a decrease of 339 articles over the same time period, the 2022 results signal that submission levels are picking up and indeed there was a 4% rise in submission over the second half of the year. Of the 2022 submissions, the proportion rejected (74%) was identical to the previous year. So far, 475 articles have been accepted for publication and of these 232 are open access (OA), a significant increase over 2020 which is encouraging. Our target for OA content this year is 48.2% of published articles. The continuing upward trend is in line with our expectation to make the transition to fully OA for *Proceedings B* by 2026. Currently OA content is downloaded about three times more often than subscription content in the journal. Our times (in median days) to first decision (24), final decision (70) and from submission receipt to publication (98) have all increased somewhat, with our times to first and final decision below our targets. It has been notable this year that many editors, authors and reviewers have requested more time for their assigned tasks resulting in slower times overall. These delays may be partly associated with Covid, but also because of work overload as research and teaching activities expand again after the pandemic. The journal's impact factor rose in 2022 to 5.530 from 5.349 the preceding year. *Proceedings B* is currently ranked 19th out of 94 journals in the Journal Citation Reports category for ‘Biology’.

We receive submissions from numerous geographical regions, with the largest numbers coming from the USA (483), China (272), UK (239), Germany (120), Australia (109), Canada (101), Japan (83), France (75), Spain (51) and Sweden (42). These rankings are quite similar to last year, although China has now replaced the UK as the country with the second most submissions to the journal. We have continued efforts to attract submissions from under-represented regions and below I discuss our initiatives to increase diversity at *Proceedings B* which we hope will help to stimulate these submissions further.

Review articles are published in most issues of *Proceedings B* and provide an outstanding forum for timely overviews of emerging areas and unresolved questions in biology. Reviews Editor Innes Cuthill has worked hard during the year to maintain a steady flow of submissions on diverse topics. To date he has received 73 proposals of which 54 have been submitted and 19 are published. Our biannual review by the *Canadian Society for Ecology and Evolution* President's Award Winner will be published shortly. The article by Lenore Fahrig and colleagues [1] addresses the common assumption that species with weak dispersal powers are likely to be at risk from anthropogenic disturbance associated with land-use intensification. This view has led to the conclusion that these species should be especially prioritized for conservation management. The authors reviewed numerous empirical studies to evaluate this and found that in many cases, the assumption does not hold and that strong dispersers are often more likely to be vulnerable than weak dispersers. In cases where the authors did find evidence for the prediction that weak dispersers were more susceptible to land-use intensification, this influence varied significantly with latitude, taxon under study and the type of anthropogenic disturbance. The authors argue that weak dispersal ability is not an appropriate general indicator of species vulnerability in human-modified landscapes.

The annual Darwin review in *Proceedings B*, ‘Theoretical studies of diverse sexual patterns in marine animals’ [2] by Yoh Iwasa (Kyushu University) and Sachi Yamaguchi (Tokyo Woman's Christian University) uses game theory as a theoretical framework for understanding diverse reproductive systems and sex allocation patterns in marine animals including corals, barnacles and fishes. The authors make the point that while game theory models of sex allocation involving fitness maximization are invaluable for understanding general patterns of sexual system diversity, future refinement of the models for specific cases should take account of the proximate mechanisms governing constraints and that this will require information from physiological, epigenetic and molecular analyses.

Special Features in *Proceedings B* involve collections of articles on a single theme chosen by the editors based on the submission of short proposals. Susan Johnston (University of Edinburgh), Nancy Chen (University of Rochester) and Emily Josephs (Michigan State University) guest-edited ‘Wild quantitative genomics: the genomic basis of fitness variation in natural population’ [3] comprising 13 articles highlighting the new field of ‘Wild Quantitative Genomics’. In recent years, new genomic technologies have enabled unprecedented insights into a range of central problems in evolutionary genetics including: the genomic architecture of polygenic variation, genotype-by-environment interactions, genetic constraints, inbreeding depression and multivariate selection, all of which are covered in this special issue. These problems are now being investigated in a wide range of non-model animals, plants and microbes, often without known pedigrees, and this collection of articles illustrates recent progress in the field.

Lockdowns associated with the Covid pandemic have made life especially challenging for academic carers. They have often had to navigate working from home at the same time as looking after young children and elderly parents. Because of these difficulties, senior editors Loeske Kruuk (University of Edinburgh), Sarah Brosnan (Georgia State University) and Maurine Neiman (University of Iowa) were inspired to show that despite these domestic constraints, and the shrinking number of hours in a day for science, many carers continued with their academic research. This special feature ‘Despite Covid: showcasing new work in evolutionary biology from academic caregivers in the middle of the pandemic’ [4] is a collection of 20 articles led largely, although not exclusively, by women, many of whom are early career researchers. A diverse set of topics is covered in this special issue including: avian behavioural changes in response to human activity during the Covid lockdown; the evolution of host proteins associated with SARS family viruses in bats; the over-representation of females as study subjects in life-history research; and thermal niches and phylogenetic assembly of tree communities in tropical montane regions. The special issue is a striking testimony to the resilience and dedication of carers engaged in scientific research.

Commentaries in *Proceedings B* have languished over the past decade, with relatively few published each year. This article type allows authors to write about particularly interesting research recently published in the journal, highlighting both strengths and weaknesses and discussing unresolved questions and future areas that require more work. Commentaries should explain the significance of the research to non-specialists and be around 2500 words, with one figure and 15 references. In 2022, we made a special effort to increase the number of these submissions by reaching out to our editors and reviewers to identify especially novel articles published in *Proceedings B* and potential authors of commentaries. We would particularly like early career researchers to write commentaries to help them promote their own academic profiles although this forum is open to all authors. I am pleased to say that our proactivity on this front has borne fruit; so far, this year we have received 10 submissions of which seven are accepted and three are currently under review. We encourage readers of *Proceedings B* to consider contacting editors if they would like to write a commentary or know a young scientist that might be qualified and enthusiastic about taking on this task.

During 2022, we have continued our efforts to diversify the scope of *Proceedings B* in an effort to attract a wider readership. The new article types we introduced over the past few years continue to attract submissions: *Evidence Synthesis* (14 submissions and six acceptances) and *Biological Science Practices* (19 submissions and seven acceptances). I thank editors Gary Carvalho and Stephanie Meirmans, respectively, for their efforts in making these article types as successful as they are. The new subject area for research articles—*Biological Applications*—that we introduced last year focusses on the application of concepts in the biological sciences for solving problems of environmental and societal relevance. Currently this subject area accounts for 4.2% of all submissions to *Proceedings B,* probably reflecting the growth in applied research of relevance to policymakers and resource managers associated with food security, climate change and the biodiversity crisis.

At our board meeting in May, we organized a break-out session in which senior editors and staff focused on how we might increase diversity at *Proceedings B*. Although we have been very successful in changing our editorial board so it is now close to gender parity, the representation of ethnic and geographical diversity among our editors needs to be improved. In addition, we would also like to increase diversity among our authors and reviewers. How these goals might be achieved was discussed at length, and it was generally agreed that it was critical that we expand the conversation to include our Associate Editors (AEs) by holding a video workshop on the topic of increasing diversity at *Proceedings B*. The workshop was subsequently organized by Stephanie Meirmans, Maurine Neiman and Shalene Singh-Shepherd on 8 November. All AEs were invited to the workshop and an impressive number of participants (45) engaged enthusiastically via Zoom to discuss three key questions: (i) what does diversity mean to you? (ii) does diversity matter and why? and (iii) how do you envision ways in which AEs can help improve diversity? These discussions resulted in a ‘Diversity Action Plan’ which includes several initiatives. These include including an annual commitment to recruit more AEs from Africa, Asia and South America and to also expand the pool of reviewers from these regions. To assist new reviewers from developing countries, we also plan to develop several blogs on how to write a good review suitable for *Proceedings B* with examples. Fortunately, because of our adoption of the Web of Science transparent peer review system (formerly Publons Open Peer review), in which the review history of a paper is now published, this will serve as a valuable resource for inexperienced reviewers.

Our goal with the Diversity Action Plan is not to simply increase diversity for the sake of diversity, but rather to appreciate and understand the practical benefits that this is likely to bring to the journal. For example, having AEs and reviewers from diverse regions should bring fresh perspectives to *Proceedings B* and also encourage submissions from researchers in countries that only occasionally publish in the journal. Also, by expanding the global representation of our reviewer pool, we are hopeful that this will help to counter the problem of ‘reviewer fatigue’. Finding sufficient reviewers for submissions is becoming an increasing problem for many scientific journals and requires increasing numbers of referee invitations before the desired number of reviews is obtained.

Several new journal developments this year that should be of interest to our readers include increasing the length of papers and dropping print and page charges in 2023. A recent author survey indicated that many authors found the limit of 10 printed pages too low. A particular frustration was when important information had to be moved to the supplementary materials during revision because of the length limit. Most other journals operate to specific word counts rather than page limits, and it was therefore agreed at our editor's meeting that *Proceedings B* should increase the length of papers to 10 000 words. With a backdrop of increased print and postage costs, the decline in the market for print copies and the Royal Society's green agenda starting in January 2023, *Proceedings B* will no longer be in print—an historic but necessary change. From next year, the journal will also no longer require page charges for authors publishing under the non-OA model.

I extend my thanks to the 10 Senior Editors and 152 AEs for their dedication in handling in an expeditious manner the large volume of submissions we receive, evaluating reviews and making recommendations. For those who are coming to the end of their terms and are leaving us in 2023, I hope that your work with *Proceedings B* has been a valuable experience which has broadened your horizons. As I mentioned last year in my editorial, it is heartening that most individuals whom we contact about joining the editorial board accept our offer and many also agree to continue for a second term despite the numerous other work pressures that academic life brings. Over the past few months, we have recruited 29 new AEs, 44% of whom are female, thus maintaining the near gender parity on our editorial board. Among the new cohort are AEs from Costa Rica, India, Israel, Japan, Singapore and Taiwan.

To conclude, I would like to thank our editorial team in London, consisting of Editorial Coordinators Jennifer Kren and Callum Shoosmith and Production Editor Simon Clackson, for their attention to detail and conscientious work in making sure that *Proceedings B* runs efficiently and on time. Lastly, I would like to thank the Publishing Editor of *Proceedings B,* Shalene Singh-Shepherd for her support, enthusiasm and wise council. Shalene is always open to new initiatives and this year has spearheaded several new projects that are helping to transform the journal as well as mentoring our first *Proceedings B* intern—Laura Steel (Oxford University)—who has been helping us with the analysis of data submissions. I wish you all a healthy, prosperous and productive 2023 and look forward to another successful year at *Proceedings B*.
